# Utilization of Transvaginal Morcellation for Large Cervical Fibroid During Laparoscopic Assisted Hysterectomy: A Case Report

**DOI:** 10.1155/crog/5522455

**Published:** 2026-04-21

**Authors:** Apoorva Kakkilaya, Karren Lewis

**Affiliations:** ^1^ John Sealy School of Medicine, University of Texas Medical Branch, Galveston, Texas, USA, utmb.edu; ^2^ Department of Obstetrics and Gynecology, TidalHealth, Salisbury, Maryland, USA, tidalhealth.org

**Keywords:** case report, cervical fibroid, laparoscopic hysterectomy, vaginal myomectomy

## Abstract

**Introduction:**

Cervical fibroids are a rare subtype of uterine fibroid (0.6%) which may cause pelvic pain and abnormal uterine bleeding. When large, they present technical challenges to traditional laparoscopic hysterectomy due to anatomic distortion and limited uterine mobility. This report aims to illustrate a minimally invasive approach to hysterectomy complicated by a large cervical fibroid with limited mobility and laparoscopic visualization.

**Case Description:**

A 51‐year‐old G6P5015 woman underwent hysterectomy for alleviation of pelvic pain secondary to an 11 cm fibroid. Intraoperatively, the fibroid occupied the entire posterior cul‐de‐sac near the right ureter and extended into the right broad ligament with posterior displacement of the cervix. A combined laparoscopic and vaginal approach was used to complete the surgery. The fibroid was morcellated through a transvaginal approach allowing for debulking of the cervix and completion in a minimally invasive fashion. The patient had an uncomplicated postoperative course.

**Conclusions:**

The potential for laparoscopic assisted vaginal hysterectomy (LAVH) with transvaginal morcellation of fibroids as an alternative to total abdominal hysterectomy (TAH) is a worthwhile consideration to reduce recovery time and postoperative complications that can accompany TAH.

## 1. Introduction

Cervical leiomyomas, or fibroids, are a rare subtype of uterine fibroids, accounting for approximately 0.6% of all fibroids [1]. Treatment of pelvic pain and abnormal bleeding secondary to fibroids often requires surgical management. Hysterectomy for symptomatic fibroids provides the highest likelihood of definitive treatment and is often the recommended approach in women who do not desire future pregnancy [2, 3]. While laparoscopic management of uterine fibroids is well established, reports describing laparoscopic hysterectomy for large cervical fibroids remain limited. Large cervical fibroids (≥10 cm) present obstacles to laparoscopic completion due to limited mobility and anatomic distortion, rendering a high risk of bleeding or ureteral injury [4]. As a result, total abdominal hysterectomy (TAH) may be preferred in such cases [5, 6].

This study describes a laparoscopic assisted vaginal hysterectomy (LAVH) with transvaginal myomectomy for a large cervical fibroid. The operation did not require blood transfusion, and the patient recovered without complications.

## 2. Case Description

A 51‐year‐old G6P5015 woman with past medical history of hypertension and obesity (BMI 31.7) presented to clinic with chief complaints of pelvic pain, difficulty urinating, and constipation for 3 months. Her prior surgeries included three prior cesarean sections and tubal ligation.

**Figure 1:**
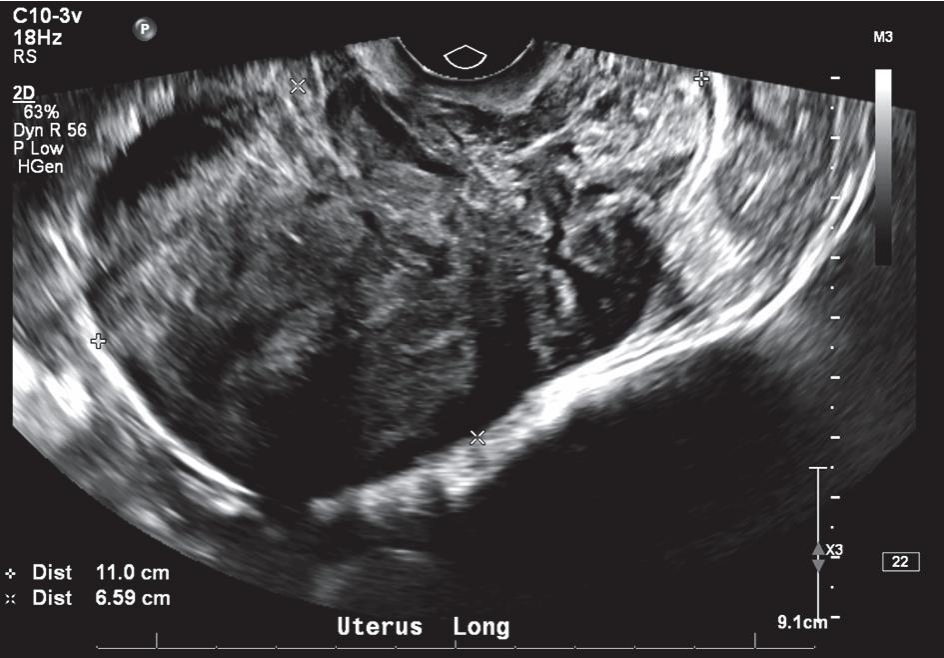
Transvaginal ultrasound image of leiomyoma.

On exam the uterus was mobile and enlarged with significant posterior bulk. The cervix and vagina were without lesions or tenderness. Transvaginal ultrasound (TVUS) demonstrated a posterior fibroid measuring approximately 11 cm (Figure 1). Endometrial biopsy and pap smear were both benign. Following thorough counseling and shared decision making, the patient desired definitive surgical management with hysterectomy. Given patient comorbidities, including three prior cesarean sections and Class 1 obesity, and single fibroid, plan was made to attempt laparoscopic surgery with possible vaginal assistance.

On intraoperative exam the large posterior fibroid was palpated in the posterior fornix. Placement of uterine manipulator was achieved in standard fashion. Laparoscopic findings included a mobile uterus extended to midpoint between umbilicus and pubic symphysis with a cervical fibroid occupying the entire posterior cul‐de‐sac and extending into right broad ligament. Retroperitoneal dissection was completed to ensure ureter location and ligate the uterine artery at its origin. After securing the uterine artery at its origin, the hysterectomy was initiated in a traditional fashion.

**Figure 2:**
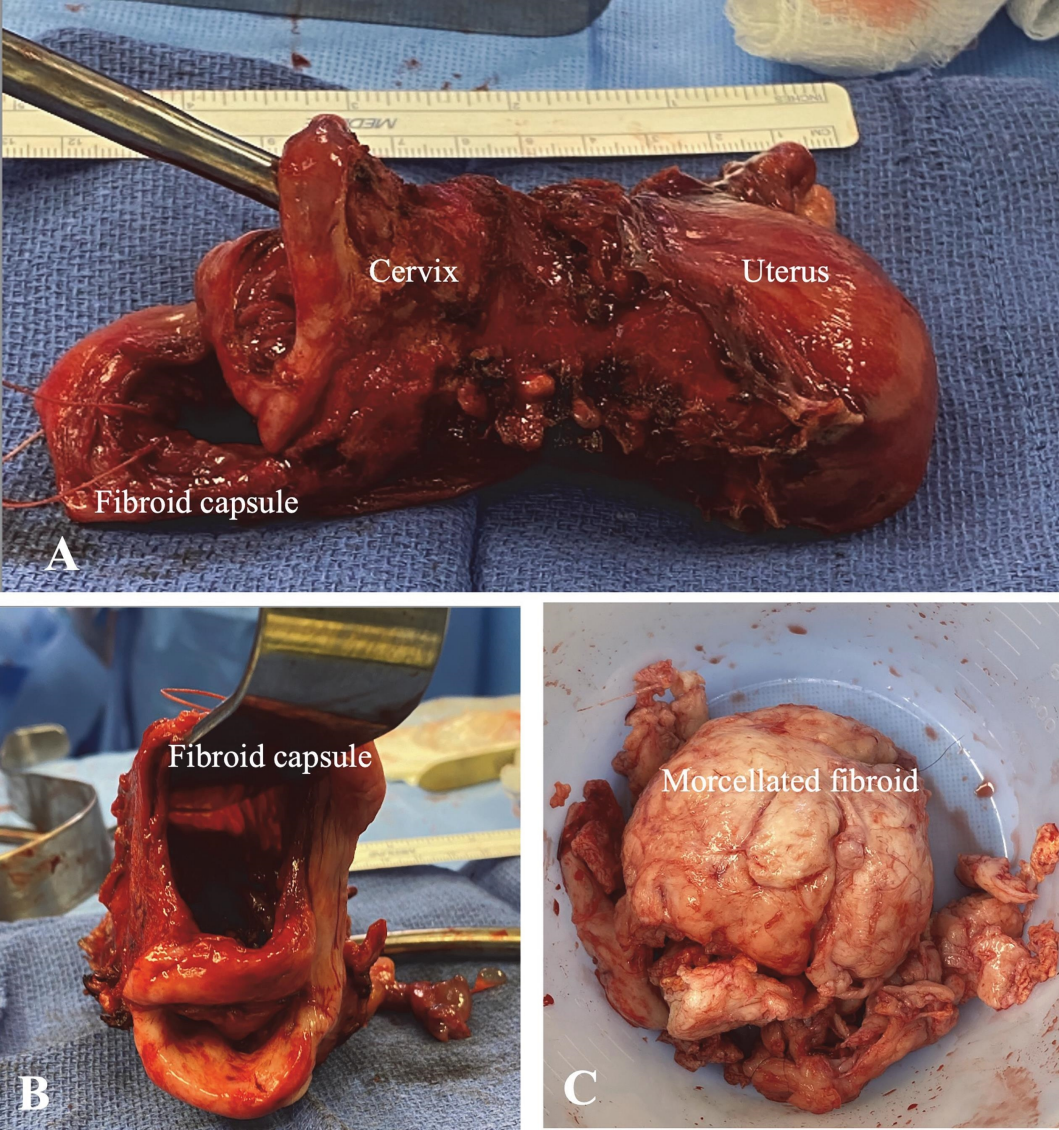
(A) Uterus, cervix, and fibroid capsule. (B) Fibroid capsule following enucleation with cervix stretched on posterior surface. (C) Fibroid specimen retrieved from transvaginal myomectomy. Excised tissue weight was 364 g.

After securing the blood supply to the uterus, anterior colpotomy was obtained with monopolar scissors. Due to the posterior bulk of the cervical fibroid with the uterus extending cephalad and anteriorly, it was not possible to antevert the uterus and fibroid sufficiently out of the posterior cul‐de‐sac to be able to continue the colpotomy posteriorly. The decision was made to proceed vaginally. Anterior colpotomy was identified and used to further delineate the remaining cervical tissue. An incision was made in the thin posterior vaginal wall under the posterior edge of the cervix. Through this incision the fibroid capsule was opened. The fibroid was then morcellated out of this incision until complete removal was completed without breaching the fibroid capsule intra‐abdominally. This allowed for safe extraction of the specimen via the vagina. Using electrocautery, the colpotomy was extended bilaterally to access the cardinal ligaments and complete the dissection of the uterus. This allowed for removal of the remaining specimen in bloc (Figure 2). The cuff was then closed with interrupted 0‐Vicryl suture, incorporating the uterosacral tissue. Cystoscopy was performed with bilateral ureteral flux and intact bladder.

Estimated blood loss was 100 mL. Patient was admitted for observation and was deemed stable to be discharged on postoperative day 1. Pathology results confirmed a benign fibroid. The patient had an uncomplicated postoperative course.

## 3. Discussion

This report illustrates a minimally invasive approach to hysterectomy when confronted with large cervical fibroids that distort the cervical and uterine cavity with no intraoperative or postoperative complications. While laparoscopy has been associated with shorter recovery times, less blood loss, and postoperative pain for hysterectomy and myomectomy with large uterine fibroids, there is currently no consensus on the optimal laparoscopic surgical approach for cervical fibroids [4].

Previous reports have described total laparoscopic hysterectomies with cervical fibroids using ureterolysis and special instrumentation such as endovascular balloon occlusion of internal iliac arteries (Table 1) [7, 8]. Total laparoscopic hysterectomy with enucleation or cervical amputation has also been described [4]. This report describes an LAVH with transvaginal morcellation for large cervical fibroids without the need for special instrumentation.

**Table 1:** Summary of reports of laparoscopic approaches for hysterectomy with cervical fibroid removal.

Author	Number of cases	Approach	Median age, years (range)	Median cervical fibroid diameter (cm) (range)	Median cervical fibroid weight (g)(range)	Reported complications
Taniguchi [4]	21	TLH: 4 patients TLH with LCA: 7 patients TLH with LECF: 7 patients TLH with both LCA and LECF: 3 patients	46 (37–65)	12 (10–16)	750 (318–1502)	1 case of ureteral injury
Nakayama et al. [7]	3	TLH with ureterolysis and transection of anterior layer of vesicouterine ligament	51 (51–59)	10.7 (6.3–10.8)	605 (501.5–1100)	1 case requiring blood transfusion intraoperatively
Takeda et al. [8]	13	LAVH with temporary endovascular balloon occlusion of bilateral internal iliac arteries	45 (40–56)	Not reported	591 (360–1010)	10 cases requiring blood transfusion intraoperatively

Abbreviations: LAVH, laparoscopic assisted vaginal hysterectomy; LCA, laparoscopic cervical amputation; LECF, laparoscopic enucleation of large cervical fibroids; TLH, total laparoscopic hysterectomy.

Several minimally invasive approaches for hysterectomy have been described for cases of large uterus, such as completion of posterior colpotomy after removal of uterine manipulator and manual intrabdominal morcellation [9]. While in‐bag morcellation offers alternative to the risks accompanied with power morcellation, it remains to be determined whether the use of a containment system eliminates the spread of tissue [10, 11]. Manual and contained techniques for transvaginal morcellation have also been described as an effective technique for large uterus when appropriate precautions are taken [12, 13]. Transvaginal morcellation via enucleation of the fibroid capsule as described in this report may offer an opportunity to avoid risks of spreading potentially malignant specimen by protection of the tissue capsule. While this approach did not utilize a containment system, contained transvaginal morcellation can be considered in situations where appropriate visibility and anatomic manipulation can be achieved.

Due to the proximity of cervical fibroids to uterine vessels, blood loss during surgery is a particular area of concern. This case provides example of successful vaginal myomectomy and LAVH technique without the need for transfusion by performing retroperitoneal dissection and ligation of uterine arteries at the origin. It is important to note the importance of appropriate expertise and laparoscopic technique for this procedure.

The potential for LAVH with transvaginal morcellation is a worthwhile consideration to reduce recovery time and postoperative complications that can accompany traditional alternatives such as TAH. Overall, critical aspects of this case include securing effective intraoperative bleeding control, careful management of ureteral distortion, and pursuing an alternative approach for morcellation and completion of posterior colpotomy.

## Author Contributions

**Apoorva Kakkilaya**: writing – original draft preparation, writing – review and editing. **Karren Lewis**: conceptualization, supervision, writing – review and editing.

## Funding

No funding was received for this manuscript.

## Consent

Verbal informed consent was obtained from the subject for publication of this case report and accompanying images.

## Conflicts of Interest

The authors declare no conflicts of interest.

## Data Availability

Data sharing is not applicable to this article as no datasets were generated or analyzed during the current study.
